# Metabolomics Biomarker Discovery to Optimize Hepatocellular Carcinoma Diagnosis: Methodology Integrating AutoML and Explainable Artificial Intelligence

**DOI:** 10.3390/diagnostics14182049

**Published:** 2024-09-15

**Authors:** Fatma Hilal Yagin, Radwa El Shawi, Abdulmohsen Algarni, Cemil Colak, Fahaid Al-Hashem, Luca Paolo Ardigò

**Affiliations:** 1 Department of Biostatistics, and Medical Informatics, Faculty of Medicine, Inonu University, 44280 Malatya, Turkey; hilal.yagin@inonu.edu.tr (F.H.Y.);; 2 Institute of Computer Science, Tartu University, 51009 Tartu, Estonia; 3 Central Labs, King Khalid University, AlQura’a, Abha 61421, Saudi Arabia; 4 Department of Physiology, College of Medicine, King Khalid University, Abha 61421, Saudi Arabia; 5 Department of Teacher Education, NLA University College, Linstows Gate 3, 0166 Oslo, Norway

**Keywords:** AI-driven diagnostics, AutoML in healthcare, metabolomics biomarkers, hepatocellular carcinoma detection, TreeSHAP interpretability

## Abstract

**Background:** This study aims to assess the efficacy of combining automated machine learning (AutoML) and explainable artificial intelligence (XAI) in identifying metabolomic biomarkers that can differentiate between hepatocellular carcinoma (HCC) and liver cirrhosis in patients with hepatitis C virus (HCV) infection. **Methods:** We investigated publicly accessible data encompassing HCC patients and cirrhotic controls. The TPOT tool, which is an AutoML tool, was used to optimize the preparation of features and data, as well as to select the most suitable machine learning model. The TreeSHAP approach, which is a type of XAI, was used to interpret the model by assessing each metabolite’s individual contribution to the categorization process. **Results:** TPOT had superior performance in distinguishing between HCC and cirrhosis compared to other AutoML approaches AutoSKlearn and H2O AutoML, in addition to traditional machine learning models such as random forest, support vector machine, and k-nearest neighbor. The TPOT technique attained an AUC value of 0.81, showcasing superior accuracy, sensitivity, and specificity in comparison to the other models. Key metabolites, including L-valine, glycine, and DL-isoleucine, were identified as essential by TPOT and subsequently verified by TreeSHAP analysis. TreeSHAP provided a comprehensive explanation of the contribution of these metabolites to the model’s predictions, thereby increasing the interpretability and dependability of the results. This thorough assessment highlights the strength and reliability of the AutoML framework in the development of clinical biomarkers. **Conclusions:** This study shows that AutoML and XAI can be used together to create metabolomic biomarkers that are specific to HCC. The exceptional performance of TPOT in comparison to traditional models highlights its capacity to identify biomarkers. Furthermore, TreeSHAP boosted model transparency by highlighting the relevance of certain metabolites. This comprehensive method has the potential to enhance the identification of biomarkers and generate precise, easily understandable, AI-driven solutions for diagnosing HCC.

## 1. Introduction

Hepatocellular carcinoma (HCC) is the most common kind of primary liver cancer with its global prevalence and matching death rates [1,2]. The incidence of HCC is still quite high in many distinct geographical locations, and it places a significant burden on public health systems all around the world. Although their causes are not limited, they include chronic hepatitis B and C infections, non-alcoholic fatty liver disease (NAFLD), alcoholic liver disease, and environmental carcinogens such as aflatoxins [3,4] are among the many contributing factors to their frequency. Moreover, the asymptomatic nature of early stage HCC often results in late detection, limiting therapeutic options and lowering the prognosis for affected individuals. As a result, awareness of the complex molecular mechanisms underlying HCC development and progression determines the progress of diagnostic approaches, the identification of biomarkers and treatment modalities, and finally the reduction of HCC-related morbidity and death rates [5,6]. In HCC diagnosis, biomarkers help with early detection, prognosis evaluation, therapy monitoring, and post-treatment recurrence surveillance. Although alpha-fetoprotein (AFP) is a well-known biomarker for HCC, poor sensitivity and specificity resulting from non-secretion by a considerable fraction of HCC tumor cells reduce its therapeutic utility [7,8]. Therefore, the continuing need for reliable biomarkers determines whether improving diagnostic accuracy, refining therapy choices, and allowing effective prognosis assessment of HCC are important [9,10]. In many human disorders, including cancer, metabolomics has been widely used in the hunt for biomarkers [11]. Representing the end products of intracellular activities, metabolites have significant potential as indicators of the overall physiological status and reactivity to environmental and host variables [12]. Although the enormous chemical diversity and concentration range of metabolites make measuring the amounts of all metabolites in a biological system challenging even with a single analytical method, the awareness of cancer metabolism—above somatic mutation—as a fundamental characteristic of cancer, originally articulated by Otto Warburg [13], stresses the indispensable relevance of metabolomics in exploring cancer biology and the development of HCC. Conventional research techniques face the risk of ignoring maybe better biomarkers during the search for metabolomics biomarkers, display design errors, and tend to emphasize certain molecules excessively [14,15].

Automating challenging tasks helps AutoML technologies alter machine learning (ML), thereby reducing or even eliminating the requirement for expert engagement [16]. From hyperparameter tuning to feature engineering to model selection and data preparation, ML has always included a series of specialized tasks. From the studies [17,18,19], several AutoML approaches have emerged over time to streamline these tasks. Tree-based Pipeline Optimization Tool (TPOT) automates ML model construction using genetic programming. TPOT excels at identifying suitable data preparation procedures, feature selection tactics, and ML approaches [20]. H2O AutoML also simplifies the ML process by employing supervised learning algorithms, ensemble learning techniques like stacking and boosting, hyperparameter tuning via random and grid search, and early stopping to increase prediction accuracy [21,22]. Designed on the Scikit-Learn framework, Auto-Sklearn is another fascinating tool. Auto-Sklearn automatically discovers and optimizes the most suitable ML algorithms and hyperparameters for specific datasets and workloads. By using Bayesian optimization, meta-learning, and ensemble building [23], it produces a powerful and efficient automated ML solution. These advances in AutoML mark a basic transformation in ML that will make advanced analysis more readily accessible and successful. Particularly in the field of biomedicine [24,25,26,27,28], along with ML models and pipelines are in growing demand alongside the automation of ML processes. This need has driven research into explainable artificial intelligence (XAI) techniques aimed at elucidating how ML algorithms generate predictions. One XAI technique, Local Interpretable Model-Agnostic Explanations (LIME), clarifies the model’s prediction for a specific instance by approximating a complex model with a simpler one around a given case, thereby providing local interpretation by using approximations. The local-to-global interpretation disparity is closed by Shapley additive explanations (SHAP). Combining the concepts of LIME and Shapley values, this whole framework provides a model-agnostic approach for evaluating predictions across many ML techniques [29]. Using Shapley values, SHAP explains “black-box” models and gives feature precedence by using interpretable local surrogate models and a cooperative game theory method. This method has been quite popular in medical fields like biomedicine and chemistry [30]. The goal of this study is to accurately find the metabolomics signature that separates HCC patients from cirrhotic (CIRR) controls using XAI and an AutoML-based algorithm. This will help find more biomarkers and learn more about the metabolic pathways that are connected to HCC. The results reveal that TPOT outperforms random forest (RF), support vector machine (SVM), and k-nearest neighbors (k-NN) among traditional ML models, and other AutoML techniques such as AutoSKlearn and H2O AutoML. This reflects how TPOT handles difficult ML tasks in terms of performance and efficiency.

## 2. Materials and Methods

**Figure 1:**
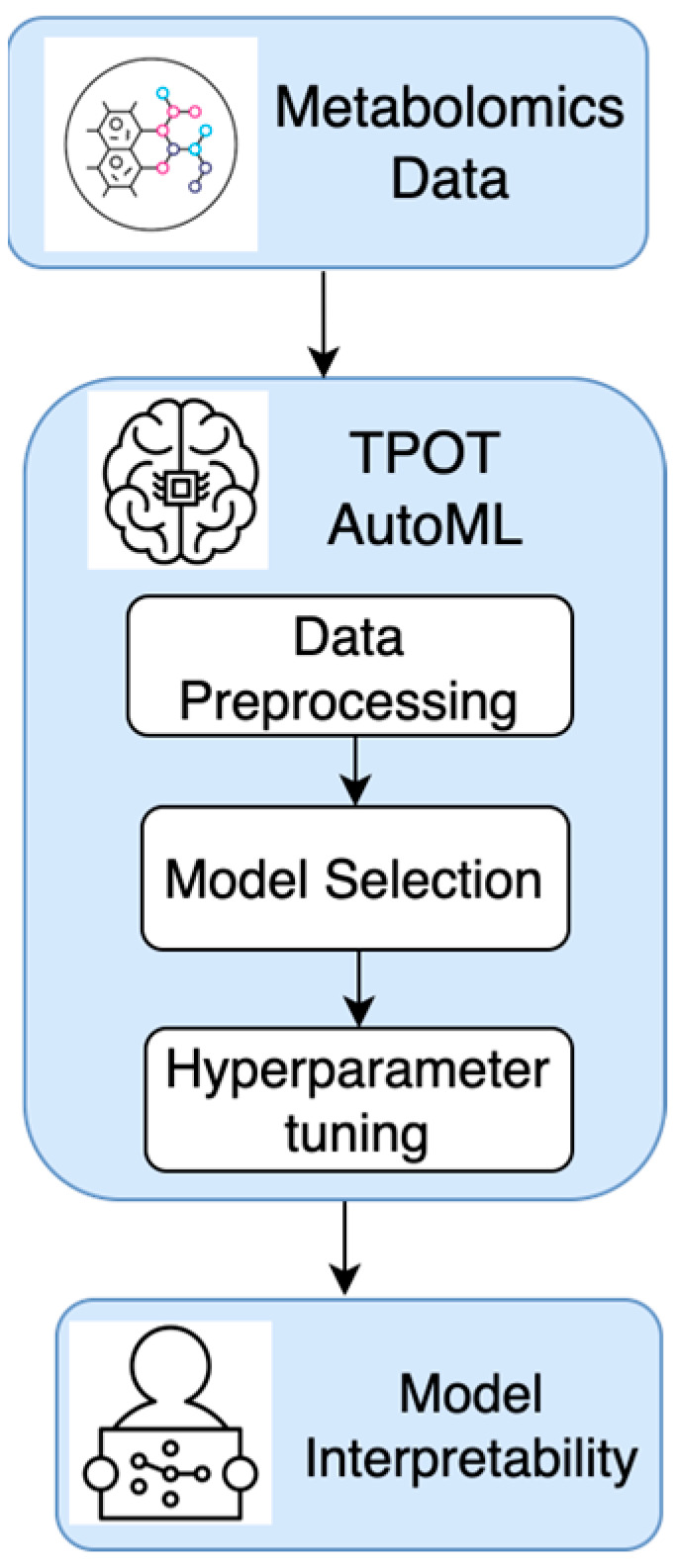
A diagram of the proposed method in the current research.

A diagram of the proposed method is provided in Figure 1.

### 2.1. Subjects, Data, and Features

In this study, we exploited a freely available dataset to discover metabolomics biomarkers capable of discriminating between patients with HCC and CIRR. The Inonu University Health Sciences Non-Interventional Clinical Research Ethics Committee approved this study (approval number: 2024/5902). Patients were diagnosed with liver CIRR based on known clinical, laboratory, and/or imaging criteria. In contrast, cases were recognized as HCC patients by well-documented diagnostic imaging criteria and/or histology. Control subjects were instructed to be free of HCC for at least 6 months from the study’s initiation. Relevant metabolite levels, resulting from gas chromatography coupled with selected ion monitoring mass spectrometry (GC-SIM-MS) tests performed on plasma samples from patients, were taken from previously published data sources [31,32]. For metabolomics analysis, a total of 56 metabolites were measured using GC-SIM-MS. These metabolites include amino acids (e.g., glycine, L-alanine, L-glutamic acid, L-leucine, L-phenylalanine, L-proline, L-serine, L-threonine, L-tyrosine, L-valine), sugars (e.g., D-glucose, L-sorbose, tagatose), organic acids (e.g., citric acid, D-malic acid, glyceric acid, lactic acid, linoleic acid, oxalic acid, palmitic acid, stearic acid), alcohols (e.g., 2 (3-butanediol, 2-hydroxybenzyl alcohol), sterols (e.g., cholesterol), vitamins (e.g., alpha-tocopherol), and other compounds (e.g., creatinine, ethanolamine, urea).

### 2.2. Automated Machine Learning with TPOT

The TPOT framework, established by Olson and Moore (2016) [20], leverages genetic programming to automatically design and improve ML pipelines. Inspired by the concept of natural selection, genetic programming exploits a population of candidate solutions developed across generations for better performance. Starting with a group of randomly generated pipelines, each representing a set of steps for data preparation, feature selection, and model selection, TPOT keeps testing each pipeline’s performance using cross-validation. We then select pipelines to serve as “parents” for the subsequent generation. These parent pipelines undergo genetic processes such as crossover, which merges pipeline segments, and mutation, which randomly transforms sections of a pipeline, to create “offspring” pipelines. Over several generations, TPOT iteratively improves the population of pipelines as this process of selection, crossover, and mutation unfolds. TPOT further mitigates overfitting by adopting a technique analogous to early stopping in machine learning. It checks pipeline performance improvements and halts the evolutionary process if no significant progress is noticed over a specific generation’s worth. Moreover, TPOT’s optimization strategy achieves a compromise between using the most effective pipelines identified so far and investigating fresh pipeline designs. One of TPOT’s key benefits is its ability to identify sophisticated pipeline designs that may not be immediately apparent through human modification. This automated strategy not only saves time but also augments the possibility of producing highly performing pipelines that successfully generalize to unknown data. TPOT makes ML pipeline design accessible to users without extensive knowledge, by automating the end-to-end ML process and providing a robust and efficient solution approach [33,34,35].

### 2.3. Model Explanation Using TreeSHAP

To interpret the model and understand the contributions of individual metabolites to the classification, we used TreeSHAP. The TreeSHAP framework leverages the hierarchical structure of tree-based models to provide efficient and consistent explanations for model predictions [36]. TreeSHAP is designed specifically for decision trees, random forests, and gradient-boosted trees, exploiting their structure to streamline the computation of Shapley values, which measure the contribution of each feature to a particular prediction. Shapley values are formally represented as:

$$\varphi_{j\left(val\right)=\frac{1}{N!}\sum_{S\subseteq N\{j\}}\left|S\right|!\left(\left|N\right|-\left|S\right|-1\right)!\left.\left[val\left(S\cup \{j\}\right)-val\left(S\right)\right.\right]}$$

TreeSHAP begins by calculating exact Shapley values for each feature in a tree ensemble model. Cooperative game theory-derived Shapley values represent the average marginal contribution of a feature across all possible subsets of features. TreeSHAP can quickly find these values by using the internal structure of tree models. This makes it much easier on computers than model-agnostic SHAP methods. One of the primary breakthroughs of TreeSHAP is its ability to perform both classification and regression tasks while preserving consistency and local correctness in its explanations. Consistency assures that if a model changes such that a specific feature’s contribution to a prediction rises or stays the same, the Shapley value for that feature will not drop. Local accuracy ensures that the sum of the Shapley values for all characteristics matches the model’s prediction for a given occurrence. TreeSHAP’s efficiency and accuracy make it especially beneficial in sectors where interpretability is critical, such as healthcare, banking, and regulatory contexts. By giving clear and accurate explanations for individual forecasts, TreeSHAP helps decision makers understand how characteristics impact model results, identify major drivers of predictions, and spot possible biases. This degree of openness promotes confidence in the model and facilitates informed decision-making. Furthermore, TreeSHAP promotes both local and global interpretability. Locally, it explains individual predictions by assigning contributions to each feature, whereas globally, it combines these contributions to give insights into the model’s general behavior. This dual feature makes TreeSHAP a flexible tool for thorough model interpretation, enabling users to get a deep grasp of both individual instances and overall patterns in the data [37,38,39].

### 2.4. Machine Learning Pipeline

We randomly divided the data into a training set (80%) and a testing set (20%) to facilitate model training and assessment. We repeated this process 50 times to ensure the model’s robustness and to obtain unbiased prediction results. For classification tasks, we implemented ML models (RF, SVC, and k-NN) from the Scikit-learn package (version 0.24.2) and AutoML methods using AutoSKlearn, H2O AutoML, and TPOT (version 0.12.2). We assessed the model performance using the Receiver Operating Characteristic Area under the Curve (ROC AUC), accuracy, sensitivity, and specificity. We allocated 600 s to the TPOT to identify suitable ML pipelines for the dataset. We used cross-validation with default settings and a hold-out method, dividing the training data into 67% for training and 33% for validation. We performed the ensemble approach, considering up to 50 models for inclusion. Each model has a runtime restriction of 240 s. We fitted the final ensemble to the whole training dataset using 5-fold cross-validation. For the XAI study, we employed SHAP (version 0.41.0) [40].

### 2.5. Statistical Analysis

Quantitative data are summarized using the median, which is represented by the interquartile range (IQR). The statistical techniques used in the univariate analysis were carefully chosen to guarantee the strength and precision of the results. At first, the Shapiro–Wilk test was used to evaluate the normality of the data distribution, which is an important step in deciding which statistical tests to use. Due to the data not following a normal distribution, the Mann–Whitney U test was used since it is a non-parametric test that is particularly suitable for assessing differences between two independent groups. This test was especially chosen for its capacity to handle data that are not distributed regularly and to provide dependable insights on differences in medians across the groups. The use of the Mann–Whitney U test, in conjunction with the criterion of considering *p*-values below 0.05 as statistically significant, highlights the meticulousness employed in examining the metabolite levels between the HCC and cirrhosis groups. The document attempts to improve the transparency and interpretability of the statistical analysis by giving this background. This will ensure that the findings are clear and methodologically sound. The statistical analyses were conducted using IBM SPSS Statistics for Windows version 26.0 software.

## 3. Results

### 3.1. Univariate Analysis Results

In the comparative analysis between CIRR and HCC groups, the following metabolites show significant differences in their median values and IQR. D-threitol is significantly lower in the HCC group (median 654203.5, IQR 405024) compared to the CIRR group (median 1000500, IQR 1225931), indicating a marked reduction. Glycine also shows a significant decrease in the HCC group (median 7312240, IQR 2826197.5) compared to the CIRR group (median 9695280.5, IQR 4818167). L-alanine-2,3,3,3-d4 is lower in HCC (median 663959, IQR 978040) than in CIRR (median 1054093, IQR 1392805.5). Conversely, L-glutamic acid-2,3,3,4,4-d5 2 is significantly lower in HCC (median 1010110, IQR 421477) compared to CIRR (median 1178557.5, IQR 414761). L-pyroglutamic acid/glutamic acid is reduced in the HCC group (median 5370125.5, IQR 2845517.75) compared to CIRR (median 6786754.5, IQR 2598769). L-valine 1 shows a significant decrease in HCC (median 3654925, IQR 3262877.5) versus CIRR (median 2502653.5, IQR 2499262.75). Linoleic acid is significantly higher in the HCC group (median 9848396, IQR 9099414.25) compared to CIRR (median 5677306.5, IQR 7210283.5). Phenylalanine 1 is significantly lower in HCC (median 1493905.5, IQR 894662.75) than in CIRR (median 2002936, IQR 1742651). Lastly, tagatose 1 is lower in HCC (median 1204101, IQR 2889017.25) compared to CIRR (median 3312035, IQR 16452131) (Table 1).

### 3.2. Model Evaluation and Performance

We compared the performance of the RF, SVM, and k-NN algorithms to AutoML using TPOT on a test dataset. TPOT significantly outperformed the baseline models, achieving an AUC score of 0.81, an accuracy score of 0.85, and a sensitivity score of 0.84. In contrast, the RF model achieved an AUC score of 0.70, an accuracy score of 0.72, and a sensitivity score of 0.71. The SVM model recorded an AUC score of 0.68, an accuracy score of 0.70, and a sensitivity score of 0.69. The k-NN model achieved an AUC score of 0.65, an accuracy score of 0.68, and a sensitivity score of 0.67. Overall, TPOT exhibited significantly superior classification performance across all metrics compared to the baseline models (Table 2). We compared the performance of TPOT with other AutoML techniques, including AutoSklearn [41] and H2O AutoML [21], using the same time budget allocated to TPOT. AutoSklearn uses Bayesian optimization to optimize pipelines built with SCIKIT-LEARN [42]. It incorporates a warm-start mechanism via meta-learning, initiating pipeline searches with the best-performing pipelines for similar datasets [43]. After the search, AutoSklearn constructs an ensemble from the trained pipelines, as described by Caruana et al. [44,45]. H2O AutoML, built on the H2O platform, performs random searches and uses custom algorithm configurations with early stopping for efficiency. It allocates more optimization time to complex algorithms like XGBoost, creates stacked ensembles from all models or the best models, and employs predefined preprocessing strategies. H2O AutoML focuses on balancing inference speed and accuracy to produce practical models for production. The comparison of AutoML techniques reveals that TPOT consistently outperforms AutoSklearn and H2O AutoML across all metrics. While TPOT maintained strong accuracy and AUC scores on both the training and test sets, AutoSklearn and H2O AutoML exhibited slightly lower performance, with a moderate drop in accuracy and AUC on the test set compared to their training results. Despite this, AutoSklearn still showed better overall performance than H2O AutoML, which lagged slightly behind in most metrics. This suggests that while all three models are effective, TPOT offers a more robust solution.

**Figure 2:**
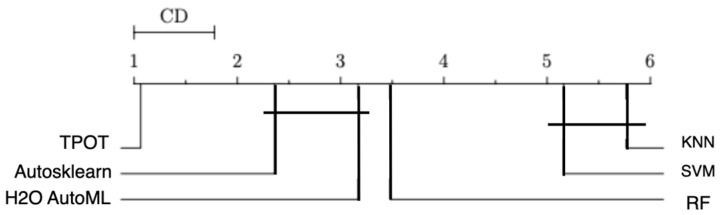
Nemenyi Test (α = 0.05) comparing the AUC of testing data for AutoML techniques and traditional machine learning techniques.

The Nemenyi test [46] was conducted to evaluate whether there are statistically significant differences in performance between TPOT, other AutoML frameworks, and traditional machine learning models, as shown in Figure 2. The results indicate that TPOT significantly outperforms the other models across several performance metrics, including AUC, accuracy, sensitivity, and specificity, with a confidence level greater than 95% (α = 0.05). Additionally, the differences between AutoSklearn, H2O AutoML, and the traditional machine learning techniques were also found to be statistically significant at the 95% confidence level. However, no statistically significant differences were observed between AutoSklearn and H2O AutoML, nor between SVM and RF.

### 3.3. Explaining the AutoML Pipeline Ensemble Using SHAP

**Figure 3:**
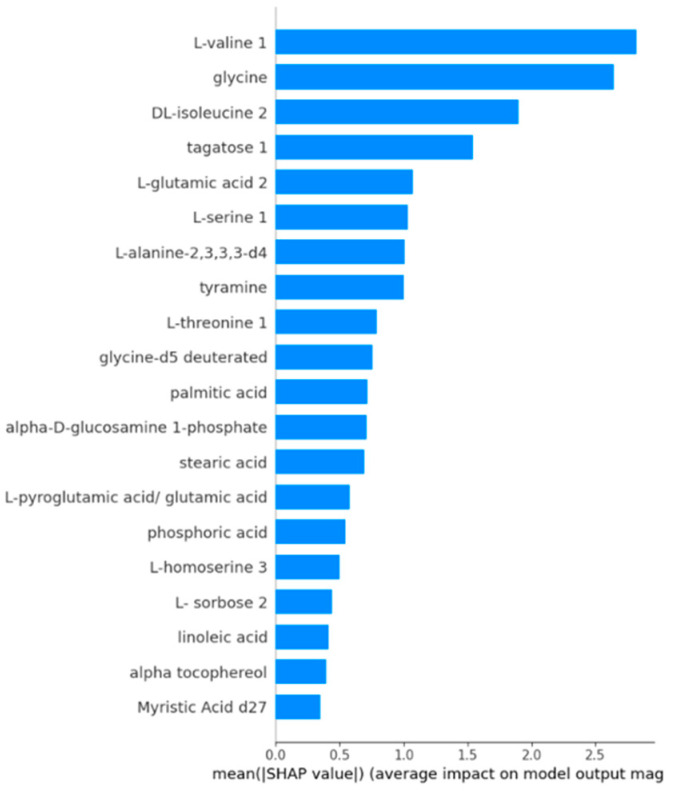
Feature importance ranking based on SHAP values.

Figure 3 presents a bar plot of feature importance based on mean absolute SHAP values for the test set, where the horizontal axis represents each feature’s average impact on the model output. The analysis reveals that L-valine 1 is the most influential feature, followed closely by glycine and DL-isoleucine 2, indicating these features significantly affect the model’s predictions. Other notable contributors include tagatose 1, L-glutamic acid 2, and L-serine 1. In contrast, features such as Myristic Acid d27 and alpha-tocopherol exhibit minimal impact on the model’s output. This ranking of features by SHAP values underscores their relative importance and enhances our understanding of the model’s decision-making process (Figure 3).

**Figure 4:**
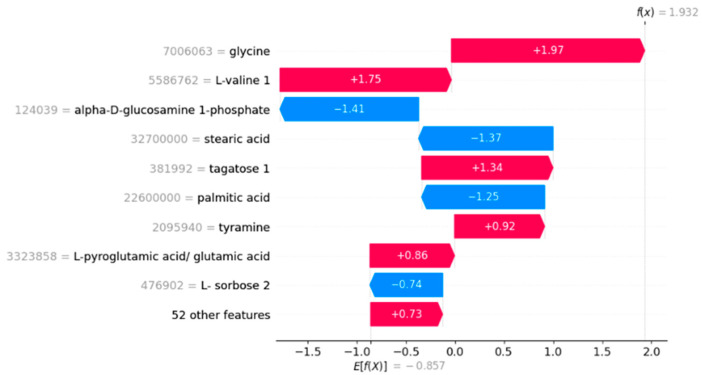
SHAP waterfall plot for a representative true positive sample.

**Figure 5:**
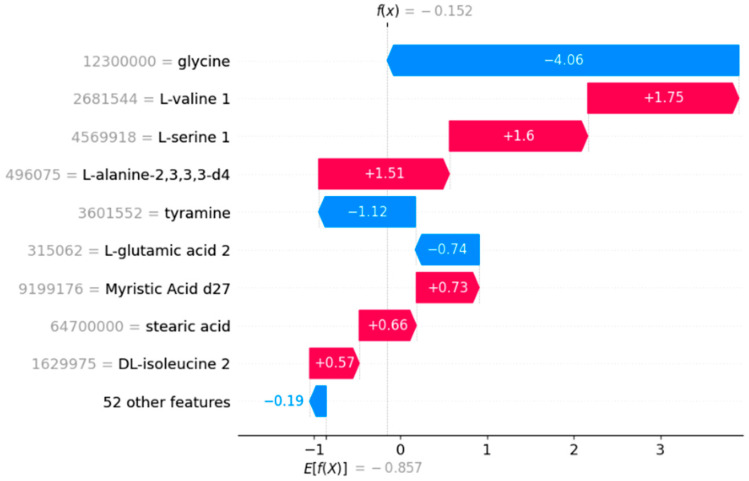
SHAP waterfall plot for a representative true negative sample.

The SHAP waterfall plots in Figure 4 and Figure 5 illustrate the feature contributions to the model’s predictions for a representative true positive and a true negative sample, respectively. In Figure 4, the base value, representing the average model output across all samples, is −0.857. The final prediction for the true positive sample is 1.932. The plot shows that features such as glycine and L-valine 1 have substantial positive contributions of +1.97 and +1.75, respectively, which drive the prediction towards the positive class. Other features like tagatose 1 and tyramine also positively contribute to the prediction, while features like alpha-D-glucosamine 1-phosphate and stearic acid have notable negative contributions of −1.41 and −1.37, respectively (Figure 4 and Figure 5).

In Figure 5, for the true negative sample, the base value is again −0.857, with a final forecast of −0.152. Glycine contributes strongly to the unfavorable prediction with a SHAP score of −4.06. Despite positive contributions from L-valine 1 and L-serine 1, which contribute +1.75 and +1.60, respectively, the negative contributions from glycine and other characteristics exceed these benefits, resulting in the overall negative forecast. These graphs give a clear insight of how various attributes impact the model’s predictions, illustrating the relative relevance and direction of each feature’s contribution in the context of particular cases.

**Figure 6:**
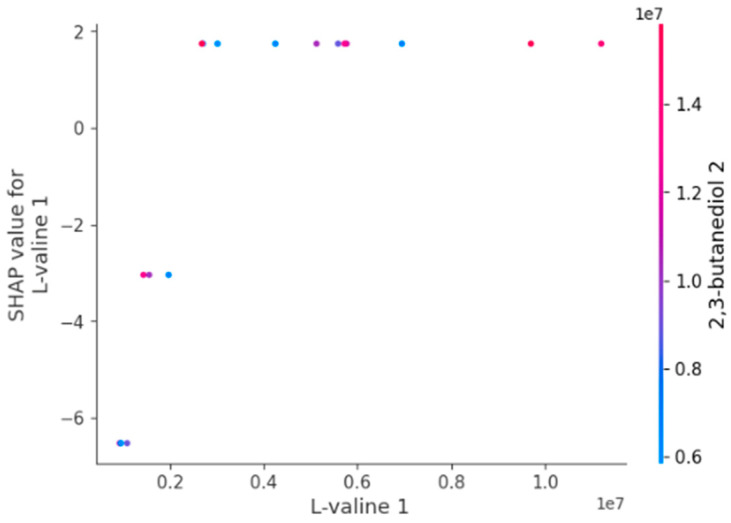
Partial dependence plot of L-valine 1 showing its SHAP value and interaction with 2,3-butanediol 2.

Figure 6 depicts the link between L-valine 1 and its SHAP value, indicating the significance of this characteristic on the model’s prediction. The x-axis displays the values of L-valine 1, while the y-axis shows the associated SHAP values, which reflect the contribution of L-valine 1 to the prediction. Notably, the figure indicates a clear shift from negative to positive SHAP values when L-valine 1 grows, indicating its dual effect on the model’s output. Furthermore, the color gradient, which depicts the values of 2,3-butanediol 2, emphasizes an interaction effect where varying amounts of 2,3-butanediol 2 regulate the effects of L-valine 1. This interaction is present throughout the spectrum of L-valine 1 values, showing a complex interplay between these factors in shaping the prediction. The strong separation of positive and negative contributions, together with the interaction effect, makes this figure especially illuminating of the underlying dynamics in the model (Figure 6).

## 4. Discussion

This investigation proposes a novel approach that leverages AutoML in conjunction with XAI techniques. The goal of this combined framework is to find metabolomics biomarkers that can tell the difference between HCC and liver CIRR in people who have been infected with HCV. Our findings demonstrate that the synergistic application of TPOT and TreeSHAP leads to substantial advancements in both the performance and interpretability of the proposed methodology. This, in turn, underscores the efficacy of the discovered biomarkers in accurately distinguishing HCC.

### 4.1. Model Performance and Interpretability Based on Metabolomics Biomarker Discovery

An AutoML-based approach was used along with XAI techniques to improve the discovery of metabolomics biomarkers that differentiate HCC from liver CIRR in people who have been infected with HCV. The TPOT demonstrated a significant improvement in model performance compared to traditional ML models such as RF, SVM, and k-NN. Specifically, TPOT achieved an AUC of 0.81, significantly outperforming RF, SVM, and k-NN, which attained AUC scores of 0.70, 0.68, and 0.65, respectively. This result underscores the efficacy of AutoML in automating the identification of optimal model configurations and relevant features, thereby enhancing diagnostic accuracy without necessitating extensive manual tuning [2,13]. The results show that TPOT achieves significantly better performance than other models in detecting HCC. We also compared the performance of TPOT with other AutoML techniques such as AutoSklearn and H2O AutoML. The comparison of AutoML techniques reveals that TPOT consistently outperforms AutoSklearn and H2O AutoML in all metrics. However, AutoSklearn showed better overall performance than H2O AutoML, which was slightly behind in most metrics. This shows that although all three models are effective, TPOT provides a more robust solution.

These findings are consistent with other studies highlighting the limitations of traditional biomarkers such as alpha-fetoprotein (AFP), which show low sensitivity and specificity. The better performance of TPOT shows that AutoML can make diagnostics more accurate by finding more relevant features and the best model configurations without a lot of manual tuning. Additionally, TreeSHAP for model interpretability provided detailed insights into the contribution of metabolites to the model’s predictions. This aspect of our study is particularly important as it addresses the need for XAI in biomedicine and enables clinicians to understand the rationale behind model predictions. Furthermore, our study identifies features like L-valine 1 and glycine as significant contributors, aligning with prior research that emphasizes the significance of amino acids in cancer metabolism.

### 4.2. Comparison with the Previous Literature

Previous studies have demonstrated the usefulness of metabolomics in cancer biomarker discovery, but they have often relied on traditional ML models such as RF, SVM, and k-NN with manual feature selection and model tuning, leading to possible biases and suboptimal performance. A paper [31] determined metabolomics profiles for HCC using traditional ML techniques but highlighted limitations due to the complexity and manual intervention required in the modeling process. In contrast, our study used TPOT, an AutoML tool that automates the creation of ML pipelines, thus reducing the manual effort and potential biases found in traditional approaches. TPOT’s genetic programming approach enables it to explore a wide range of pipeline configurations and optimize the entire process, including data preprocessing, feature selection, and model selection. This automated method has been shown to outperform traditional ML models such as RF, SVM, and k-NN and other AutoML models in terms of multiple performance metrics (AUC, accuracy, sensitivity, and specificity). The metabolites L-valine, glycine, and DL-isoleucine have been extensively studied in relation to HCC. Studies have shown that serum concentrations of L-valine, glycine, L-isoleucine, and D-isoleucine are significantly reduced in HCC patients compared to healthy individuals [47]. In addition, amino acid profiling in HCC tumor tissues revealed significant upregulation of essential amino acids, including leucine, valine, and tryptophan, suggesting their potential as metabolic biomarkers for HCC [48]. Furthermore, metabolomics analysis of serum samples from HCC patients identified glycine as one of the upregulated metabolites associated with the disease and demonstrated its potential role as a biomarker for HCC [49]. These results show that L-valine, glycine, and DL-isoleucine metabolites have a complex relationship with the development of HCC. They also show how important these metabolites are in the metabolic changes that happen in liver cancer. Researchers have studied L-valine, a branched-chain amino acid, in relation to HCC. In rats with CCl4-induced liver injury, studies have shown that L-valine treatment can improve liver fibrosis and increase thrombopoiesis. Furthermore, metabolomics studies have identified L-valine as a significant metabolite in HCC patients, with higher levels detected in HCC tumor tissues than in non-tumor tissues and in HCC patients’ serum before hepatectomy. These results point to a possible link between L-valine levels and HCC, showing that it plays a part in metabolic pathways linked to the growth and spread of HCC. Further research into the specific mechanisms underlying death may provide valuable information for developing new therapeutic approaches for this challenging cancer [48,50]. Studies have shown that changes in amino acid levels, including L-glutamine, can affect the proliferation of HCC cell lines, with supplementation or deprivation of specific amino acids leading to antiproliferative effects and changes in critical signaling molecules [51].

Previous studies have underscored the utility of metabolomics in cancer biomarker discovery. However, they have often relied on traditional ML models, which require extensive manual feature selection and tuning, potentially leading to biases and suboptimal performance [14,31]. A research paper [31] utilized traditional ML techniques to determine metabolomics profiles for HCC but faced challenges related to the complexity and manual intervention required. Our study addresses these limitations by employing TPOT, which automates the entire ML pipeline, thus reducing manual effort and potential biases [20]. The metabolites identified in this study, such as L-valine, glycine, and DL-isoleucine, have been previously associated with HCC. Serum concentrations of these amino acids are significantly reduced in HCC patients compared to healthy individuals, and their upregulation in HCC tumor tissues suggests their potential as metabolic biomarkers. These findings are consistent with our results, which highlight the importance of amino acids in cancer metabolism and their potential role as biomarkers for HCC [5,12,31].

### 4.3. Clinical Implementation, Potential Contributions, and Future Directions

The identification of L-valine, glycine, and DL-isoleucine as key metabolites offers promising avenues for clinical application. Clinical workflows can integrate these biomarkers to improve the early detection and prognosis of HCC in patients with liver CIRR and HCV infection. Moreover, TPOT’s capability to process complex datasets and automatically optimize model pipelines underscores its potential as a valuable tool for ongoing biomarker discovery efforts. Our study also emphasizes the broader applicability of AutoML and XAI techniques in medical research. The interpretability provided by TreeSHAP can enhance trust in AI models among healthcare professionals, facilitating their adoption in clinical practice. TreeSHAP meets the important need for clarity in biomedical AI applications by showing how different metabolites affect the model’s predictions [36].

Integrating metabolomics indicators including L-valine, glycine, and DL-isoleucine into clinical practice shows potential for enhancing the early identification and treatment of HCC in patients with liver cirrhosis and HCV infection. The biomarkers have shown promise in differentiating HCC from liver cirrhosis, hence assisting in identifying individuals with a greater likelihood of HCC development [48]. Nevertheless, the transition of these indicators from the realm of research to practical use in clinical settings requires a thorough strategy that addresses several obstacles. An essential obstacle is the need for thorough validation across a wide range of patient groups. The performance of biomarkers may be influenced by variables such as ethnicity, concomitant diseases, and environmental effects. Hence, it is essential to conduct extensive, multicenter investigations to authenticate these biomarkers across diverse demographic and clinical scenarios, hence ensuring their reliability and applicability. Moreover, the creation of standardized, high-throughput tests is crucial for facilitating the use of these biomarkers in regular clinical environments. The assays need to be economically efficient, dependable, and able to work with existing diagnostic platforms in order to encourage their extensive use [47]. An additional crucial factor is the incorporation of these biomarkers into current therapeutic processes. Successful implementation of this procedure requires cooperation among academic institutions, healthcare providers, and industry partners to create diagnostic tools that can easily integrate into clinical practice. Moreover, it is important to provide training to healthcare workers about the analysis and understanding of metabolomics data. Given that metabolomics is a nascent discipline in clinical diagnostics, it will be imperative to educate doctors on the optimal use of these biomarkers to ensure their successful integration [50]. It is crucial to recognize the potential of these biomarkers to not only improve early diagnosis but also guide treatment options and track therapy responses. The biomarkers might enhance individualized treatment approaches and enhance patient outcomes by offering a more comprehensive knowledge of the metabolic changes linked to HCC. Although there are difficulties, integrating these metabolomics indicators into clinical practice is a noteworthy advancement in combating HCC. This has the potential to greatly enhance the prognosis and survival rates of afflicted patients [12].

Combining genetics and proteomics with metabolomics provides a more thorough method for comprehending HCC. Using a multi-omics approach, it is possible to uncover relationships between genetic variants, protein expression, and metabolic alterations, thereby enhancing the identification of reliable biomarkers. Nevertheless, the difficulties lie in effectively handling extensive and varied information, as well as ensuring interoperability across various omics platforms. Although there are challenges to overcome, using a multi-omics approach has the potential for discovering biomarkers that are more accurate in predicting outcomes and creating tailored treatment plans.

The new framework in this study combines automated machine learning and XAI to make it easier to find metabolomics biomarkers that can help diagnose HCC. Our system differs from previous models by using the TPOT tool to automatically optimize model selection, feature engineering, and hyperparameter tuning instead of relying on standard machine learning techniques that need human feature selection. This automated procedure improves model performance, as evidenced by an improved AUC score of 0.81 compared to conventional methods, while also mitigating any biases associated with human intervention. Additionally, our method uses the TreeSHAP method to give clear and understandable details about how different metabolites, like L-valine, glycine, and DL-isoleucine, help with the diagnostic process. Our framework stands out from previous models due to its dual approach, which improves diagnosis accuracy and provides thorough knowledge of the metabolic pathways associated with HCC. This distinguishes our framework from other models that do not possess the same level of depth and interpretability. Additionally, the use of AI and AutoML for biomarker identification in HCC requires addressing key ethical concerns. Ensuring data privacy through anonymized datasets and mitigating biases in model predictions are essential to maintain fairness and integrity in clinical diagnostics.

## 5. Limitation

Although our study demonstrates the advantages of using AutoML and XAI techniques, there are limitations that need to be considered. The specific patient cohort and metabolomics profiling methods used limit the generalizability of our findings. Future research should investigate the application of these techniques in different populations and using a variety of metabolomics technologies. Additionally, combining other omics data, such as genomics and proteomics, with metabolomics may provide a more comprehensive understanding of HCC pathogenesis and lead to the discovery of multi-omics biomarkers. This integrative approach combined with AutoML can further increase the accuracy and utility of biomarker-based diagnoses. Not only that, but different metabolomics technologies, like LC-MS/MS or NMR, might produce various sets of biomarkers. This could affect how easily our results can be repeated in different clinical settings. While this study focused on optimizing traditional ML models using AutoML integrated with XAI, we plan to incorporate advanced deep learning models in subsequent work to further validate and enhance the findings. Additionally, we acknowledge that our exploration of XAI techniques was limited to TreeSHAP. Future work should consider employing other XAI methods, such as LIME or DeepSHAP, to provide a broader perspective on model interpretability and to ensure that the insights gained are consistent across different interpretability frameworks.

## 6. Conclusions

It may be argued that AutoML and XAI approaches should be implemented within this study to discover the metabolomics biomarkers for HCC. Thus, the researchers, with the help of TPOT, a tool for automated, optimal, and efficient model identification, obtained improved results in comparison with other static models when it came to distinguishing between patients with HCC and liver CIRR in individuals with HCV infection. This achievement suggests that AutoML could be effective at simplifying biomarker identification, as well as potentially exposing much more lasting diagnostic patterns. Furthermore, the study used TreeSHAP, an XAI method, to explain the model’s inner workings to analysts. This also provided insight and an estimate of each metabolite’s contribution to the classification procedure. Revealing these features improves clinical personnel’s confidence and acceptance towards AI-driven models, increasing their likelihood of deployment in clinical settings. Thus, the findings of this research provide very effective evidence of using AutoML and XAI concurrently for identifying biomarkers of HCC. This approach to fusion has a lot of potential to enhance the existing methods in AI-powered diagnostics since, rather than working in isolation, both models will be able to interact and learn from each other to improve the performance of diagnoses, thereby enhancing the notion of patient care.

## Figures and Tables

**Table 1:** Univariate statistical analysis results.

Metabolite Name *	Group		*p*-Value
	CIRR	HCC	
2,3-butanediol 2	10650000 (5477273.75)	9922545 (6109323.25)	0.519
2-hydroxybenzyl alcohol	548521.5 (175550.5)	539983.5 (132430)	0.547
alpha-tocopherol	1406009 (772343)	1671573.5 (675712)	0.075
alpha-D-glucosamine 1-phosphate	451731 (6850415.25)	1359994 (11365839.25)	0.123
arabitol	378181.5 (390092.75)	355639.5 (258970.75)	0.240
arachidic acid	418434.5 (291183.25)	330568 (430527)	0.745
cholesterol	92450000 (3.9e+07)	93400000 (32175000)	0.554
citric acid	20400000 (11675000)	18900000 (8450000)	0.128
Creatinine	1917199 (2062927)	1211467.5 (1116398.5)	0.053
D-glucose 2 [17,625]/[24,749] D-glucose 1	1.13e+08 (37175000)	111500000 (42125000)	0.785
D-malic acid	467819.5 (255174.25)	378374.5 (348014.25)	0.251
D-threitol	1000500 (1225931)	654203.5 (405024)	0.014
diglycerol 2	221418 (384361)	170021 (201316.25)	0.081
DL-isoleucine 1	1199501.5 (772889)	1433657 (1229352)	0.194
DL-isoleucine 2	1131049 (618355.25)	1424988 (1050869.25)	0.051
ethanolamine	1352473 (958388.25)	1068951 (654469)	0.075
glyceric acid	683817.5 (560978)	625615 (518581.75)	0.577
glycine	9695280.5 (4818167)	7312240 (2826197.5)	0.003
glycine-d5 deuterated	23850000 (10650000)	21500000 (12225000)	0.293
L-sorbose 2	1185333 (1842600.25)	1185925 (1437230.75)	0.596
L-(+) lactic acid	94950000 (48875000)	95500000 (44650000)	0.594
L-alanine-2,3,3,3-d4	1054093 (1392805.5)	663959 (978040)	0.013
L-cystine 3	2434071 (2331622)	2399262.5 (2699936.75)	0.793
L-glutamic acid 2	384084.5 (364430.5)	357128.5 (432405)	0.685
L-glutamic acid-2,3,3,4,4-d5 2	1178557.5 (414761)	1010110 (421477)	0.047
L-glutamic acid-2,3,3,4,4-d5 3 (dehydrated)	1879400 (698975.25)	1539727 (1043167)	0.351
L-homoserine 3	73072.5 (76962.5)	57437 (43002.75)	0.383
L-leucine 1	3958303 (3744917.75)	4579521 (4642521.5)	0.091
L-phenylalanine-phenyl-d5-2,3,3-d3 2	5153990.5 (2038524.25)	4548072 (2141348.5)	0.144
L-proline 2	2773618 (1277443)	2310765 (1221758)	0.209
L-pyroglutamic acid/glutamic acid	6786754.5 (2598769)	5370125.5 (2845517.75)	0.007
L-serine 1	2358091 (1530538.25)	2598062 (1569120.25)	0.380
L-threonine 1	3226459 (1786736.5)	3165103 (1790904)	0.904
L-threonine 2	4140405.5 (2479098.5)	3530755 (2347793.5)	0.365
L-tyrosine-3,3-d2 2	25400000 (9275000)	25950000 (15525000)	0.868
L-valine 1	2502653.5 (2499262.75)	3654925 (3262877.5)	0.008
L-valine 2	2380131.5 (2110335.5)	2615713 (2691378.5)	0.425
lactulose 1	223597 (614729)	186756.5 (535900.75)	0.621
lauric acid	252586.5 (223034.5)	205633.5 (197828.75)	0.170
linoleic acid	5677306.5 (7210283.5)	9848396 (9099414.25)	0.006
myo-inositol	8412012 (10620568.5)	7559708 (5249310)	0.613
Myristic Acid d27	6418506.5 (2757576.75)	6669544 (3364288.5)	0.634
N-acetyl-5-hydroxytryptamine 1	105500000 (21350000)	104500000 (45800000)	0.968
oxalic acid	28250000 (16350000)	31300000 (16975000)	0.322
palmitic acid	27600000 (13550000)	31750000 (16125000)	0.179
Phenylalanine 1	2002936 (1742651)	1493905.5 (894662.75)	0.040
phosphoric acid	84250000 (27750000)	82800000 (39875000)	0.610
putrescine	896244.5 (812358)	672440 (488520.75)	0.342
ribitol	634169 (501046)	487043.5 (320140)	0.097
ribose	77076.5 (78126.25)	43190 (51127.25)	0.165
stearic acid	44650000 (21650000)	49150000 (21050000)	0.182
tagatose 1	3312035 (16452131)	1204101 (2889017.25)	0.006
trans-aconitic acid	112768 (80640.5)	96625 (70570.5)	0.457
tyramine	3083548.5 (3382113.25)	1700785 (2529014)	0.095
tyrosine 2	24800000 (11975000)	24050000 (14600000)	0.872
urea	98800000 (66050000)	99850000 (60625000)	0.788

*: Variables are summarized as median (interquartile range; IQR).

**Table 2:** Comparative performance of ML models and AutoML techniques versus TPOT. Training set results are presented as scores (±standard deviation). Reported metrics include ROC AUC, accuracy, sensitivity, and specificity.

Model	Train Set AUC	Test Set AUC	Train Set Accuracy	Test Set Accuracy	Train Set Sensitivity	Test Set Sensitivity	Train Set Specificity	Test Set Specificity
TPOT	0.80 ± 0.02	0.81	0.85 ± 0.01	0.85	0.84 ± 0.03	0.84	0.85 ± 0.01	0.83
RF	0.72 ± 0.02	0.70	0.74 ± 0.03	0.72	0.72 ± 0.04	0.71	0.75 ± 0.03	0.71
SVM	0.70 ± 0.03	0.68	0.72 ± 0.03	0.70	0.70 ± 0.04	0.69	0.73 ± 0.04	0.70
k-NN	0.66 ± 0.03	0.65	0.70 ± 0.03	0.68	0.68 ± 0.04	0.67	0.72 ± 0.03	0.68
AutoSklearn	0.75 ± 0.01	0.77	0.75 ± 0.02	0.73	0.70 ± 0.02	0.74	0.77 ± 0.01	0.74
H2O AutoML	0.74 ± 0.02	0.75	0.76 ± 0.03	0.75	0.74 ± 0.03	0.73	0.77 ± 0.02	0.73

## Data Availability

The raw data supporting the conclusions of this article will be made available by the authors on request.
